# HSP-1-specific nanobodies alter chaperone function *in vitro* and *in vivo*

**DOI:** 10.1016/j.jbc.2026.111238

**Published:** 2026-02-04

**Authors:** Nicholas D. Urban, Kunal Gharat, Zachary J. Mattiola, Ashley Scheutzow, Adam Klaiss, Sarah Tabler, Asa W. Huffaker, Monique Grootveld, Mary E. Skinner, Weiyang Zheng, Matthew J. O’Meara, Janine Kirstein, Matthias C. Truttmann

**Affiliations:** 1 Department of Molecular & Integrative Physiology, University of Michigan, Ann Arbor, Michigan, USA; 2 Leibniz Institute on Aging - Fritz-Lipmann-Institute (FLI), Jena, Germany; 3 Department of Biochemistry, University of Michigan, Ann Arbor, Michigan, USA; 4 Sanquin, CX Amsterdam; 5 Gilbert S. Omenn Department of Computational Medicine and Bioinformatics, University of Michigan, Ann Arbor, Michigan, USA; 6 Department of Medicinal Chemistry, University of Michigan, Ann Arbor, Michigan, USA; 7 Friedrich-Schiller-Universität, Institute for Biochemistry and Biophysics, Jena, Germany; 8 Geriatrics Center, University of Michigan, Ann Arbor, Michigan, USA

**Keywords:** nanobody, proteostasis, chaperone, heat shock protein, HSP70, HSC70, HSP-1, *C*. *elegans*

## Abstract

Targeted regulation of 70 kDa heat shock protein (HSP70) chaperones, particularly the essential cognate heat shock protein (HSC70) and its *Caenorhabditis elegans* ortholog (HSP-1), may hold the key to improving cellular proteostasis and ameliorating aging-associated conditions linked to protein misfolding and aggregation. However, tools to selectively alter HSP70 chaperone activity remain elusive. In this study, we pioneer the development of two novel nanobodies, B12 and H5, which specifically bind to both recombinant and endogenous HSP-1. We show that these nanobodies, differing by only two amino acids in their complementarity-determining regions, bind specifically to HSP-1 and effectively reduce both HSP-1 ATPase activity and protein folding capacity in a dose-dependent manner *in vitro*. We further demonstrate *in vivo* expression of B12, but not H5, in transgenic *C. elegans* strains reduces heat-stress survival and proteotoxic-stress resistance, mirroring the effects of *hsp-1* knockdown *via* RNA interference. Our findings suggest that these nanobodies can serve as effective and specific tools for inhibiting HSP-1 chaperone activity *in vivo*. These discoveries provide a foundation for future research exploring the therapeutic potential of HSP70-targeting nanobodies in aging and protein misfolding diseases.

The 70 kDa heat shock protein (HSP70) family consists of conserved, ATP-dependent molecular chaperones critical for maintaining cellular proteostasis during stress (1). Each monomer includes an approximately 44 kDa nucleotide-binding domain (NBD), which mediates ATP binding and hydrolysis, and an approximately 28 kDa substrate-binding domain (SBD) that contains a hydrophobic pocket for binding polypeptides (2, 3). The lid of the SBD regulates substrate interaction, with conformational changes initiated in the NBD through ATP hydrolysis, affecting the trapping of substrates in the SBD (4, 5, 6). This allosteric cycle transitions HSP70 between high- and low-affinity states for client engagement (7). Targeted interactions with co-chaperones enhance HSP70 activity and provide for greater diversification in substrate interaction. J-domain proteins interact with the NBD of HSP70s to stimulate ATP hydrolysis, while nucleotide exchange factors interact with the NBD to facilitate ADP-ATP exchange, acting as molecular adaptors in physiological contexts (8, 9, 10).

Functionally, HSP70 chaperones aid in the folding of newly synthesized proteins, prevent protein aggregation by stabilizing protein folding intermediates, refold and disaggregate misfolded and aggregated proteins, direct misfolded proteins to degradation pathways, and enable protein trafficking (6). Dysregulated HSP70 function is implicated in diseases such as Alzheimer’s disease, Parkinson’s disease, and cancer metastasis (11, 12, 13, 14, 15). With current clinical research exploring inhibitors and activators, HSP70s remain promising therapeutic targets for age-related diseases and conditions involving protein misfolding (16, 17).

The HSP70 family is highly conserved across species, highlighting their fundamental role in proteostasis (6, 18). In humans, there are 13 HSP70 genes, a figure mirrored in mice; *Caenorhabditis elegans* have about seven functional HSP70 genes, although broader classifications that include pseudogenes can raise this total to between 10 and 16 (17, 19). Heat shock cognate 71 kDa protein (also known as HSC70 or HSPA8) is the constitutively expressed HSP70 family chaperone in mammalian cells, supporting protein folding, complex assembly, and the refolding or degradation of misfolded proteins (20). In the face of cellular stress, HSC70 can translocate to the nucleus to avert aggregation of heat-denatured nuclear proteins, aiding nuclear stability (21, 22, 23, 24). In *C. elegans*, the HSC70 ortholog, HSP-1, fulfills a similar function, facilitating development, promoting resistance to stress, and regulating proteostasis by ensuring proper nascent polypeptide folding and minimizing protein aggregation (25, 26). Both homologous proteins also intersect with proteolytic pathways to maintain cellular quality control; thus, the shared roles of HSC70 and HSP-1 underscore the high degree of evolutionary conservation within the HSP70 family, affirming their significance in safeguarding organismal health and fitness across different species (1, 26, 27, 28, 29, 30). However, specific tools to modulate HSC70 and HSP-1 activity remain elusive.

In this study, we identify a pair of nanobodies which specifically bind and inhibit the activity of the cognate *C. elegans*’ chaperone, HSP-1. Nanobodies, also known as variable domain of the heavy chain (VHH), are single-domain antibody fragments derived from the heavy-chain-only antibodies found in camelids (*e.g.*, camels, llamas, and alpacas) and sharks (31, 32). Consisting solely of the variable domain, they measure approximately 15 kDa, enabling them to bind epitopes often inaccessible to larger antibodies, including enzyme active sites (33, 34). Their high specificity and affinity, combined with excellent solubility and remarkable thermal and chemical stability, make them valuable tools in both research and therapeutic contexts (35, 36). Nanobodies can be efficiently produced in bacterial systems, such as *Escherichia coli*, as well as expressed in eukaryotic systems where they retain functionality (37, 38). Given their tissue-penetration capabilities and rapid clearance from the bloodstream, nanobodies see increasing use in biosensing, as positron emission tomography (PET) tracers, and as targeting entities of PROTACs (39, 40, 41, 42).

Here, we present two nanobodies, B12 and H5, which differ in only two amino acids and bind specifically to both recombinant HSP-1 *in vitro* and endogenous HSP-1 *in vivo* in *C. elegans*. Using ATPase and protein refolding assays, we show both B12 and H5 inhibit the ATPase activity and refolding capability of HSP-1 in a dose-dependent manner. Furthermore, we find that moderate *in vivo* expression of B12 is sufficient to phenocopy reductions in survival and protein misfolding (Aβ_1-42_)-induced paralysis akin to *hsp-1* knockdown in *C. elegans*. Taken as a whole, these results establish B12 and H5 as two specific nanobodies to specifically regulate HSP-1-dependent processes *in vitro* and *in vivo*.

## Results

### Nanobodies B12 and H5 bind specifically to HSP-1 *in vitro*

**Figure 1:**
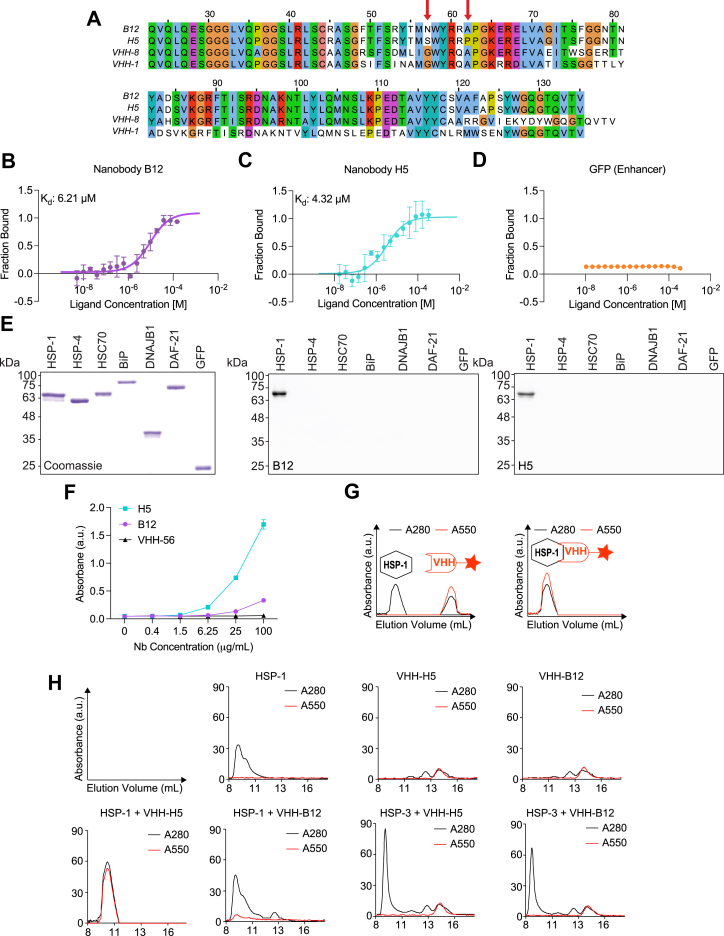
**Generation of HSP-1-specific nanobodies B12 and H5.***A*, sequence alignments of nanobodies B12, H5, and two other nanobodies (VHH-1 and VHH-8) isolated from the same immune library without HSP-1 binding activity. *Red arrows* indicate differences in two amino acids between B12 and H5. *Blue*: Amino acids with hydrophobic side chains; *Green*: Amino acids with polar uncharged side chains; *Purple*: Amino acids with negatively charged side chains; *Red*: Amino acids with positively charged side chains; *Other colors*: special cases (*B*–*D*). MST binding curves for HSP-1 (20 nM) titrated against B12 (*B*, 0.003117–102.2 μM), H5 (*C*, 0.02664–436.5 μM) and VHH Enhancer (*D*, 0.0106–350 μM). Data represent mean ± SD of three replicates. *E*, coomassie stained SDS-PAGE analysis (*left*) and western blots using B12 (*middle*) or H5 (*right*) detecting the depicted chaperones and GFP. *F*,. example ELISA using HSP-1 as the bait protein. *G*, schematic of size exclusion chromatography absorbance readouts. *H*, size-exclusion chromatography of indicated combination of protein(s) and nanobody. For western blots and ELISAs, nanobodies were detected using the Precision Protein StrepTactin-HRP Conjugate (Bio-Rad, Cat #161038). VHH, variable domain of the heavy chain; HRP, horseradish peroxidase; HSP, heat shock protein.

To generate nanobodies specific for HSP-1, we immunized an alpaca with recombinant HSP-1 protein purified from *E. coli* overexpression cultures. We then isolated peripheral lymphocytes, extracted RNA, and amplified the VHH-coding sequences to clone them into a phagemid library. Next, we performed phage display on immobilized HSP-1 to enrich for HSP-1-binding VHHs. We then tested 90 VHH clones in a crude ELISA setup, of which 13 showed strong binding. Sequencing of the binders revealed that the best performing VHHs represented two unique VHH sequences, which we named B12 and H5 (Fig. 1*A*). Interestingly, B12 and H5 differ by only two amino acids in complementarity-determining region (CDR) 1 and have identical CDR2 and CDR3 domains (Fig. 1*A*).

After cloning these sequences into an *E. coli* overexpression vector suitable for periplasmic nanobody expression (pHEN), we purified B12 and H5 and evaluated the specificity and binding capacity of each nanobody for recombinant HSP-1. Binding assays determined a K_d_ of approximately 6.2 μM for B12 to HSP-1 and a K_d_ of 4.3 μM for H5 to HSP-1 (Fig. 1, *B* and *C*). Importantly, control nanobody (anti-GFP; VHH_Enhancer_) (43) did not bind to HSP-1 (Fig. 1*D*). Next, we determined the ability and specificity of biotinylated B12 and H5 to detect HSP-1 in western blot and ELISA assays. Both nanobodies recognized HSP-1 in these assays, confirming antigen targeting *in vitro* (Figs. 1, *E* and *F* and S1, *A*–*D*)). Notably, neither B12 nor H5 detected other *C. elegans* or human HSP70 chaperones (*e.g.*, HSP-4, HSC70, and BiP), or any other non-HSP70 protein tested (*e.g.*, DNAJB1, DAF-21/HSP90, and GFP) in western blotting experiments (Fig. 1*E*). Notably, in ELISA assays, H5 demonstrated a noticeably stronger binding capability compared to B12 (Figs. 1*E* and S1*D*), aligning with the enhanced binding affinity of H5 for properly folded HSP-1 suggested by the binding kinetics experiments (Fig. 1, *B*, *C*, and *F*).

To test B12 and H5 interactions with HSP-1 in an aqueous solution, we labeled both nanobodies with a C-terminal TAMRA fluorophore using sortase technology (44) and analyzed nanobody-HSP-1 complex formation by analytic size-exclusion chromatography (Fig. 1, *G* and *H*). We observed stable binding of B12 and H5 to HSP-1 as indicated by the TAMRA signal eluting with the chaperone. Consistent with previous experiments, neither nanobody bound to HSP-3, a *C. elegans* BiP/HSPA5 ortholog (Fig. 1*H*).

These findings establish both B12 and H5 as selective binders of HSP-1, with H5 exhibiting stronger binding *in vitro*.

### B12 and H5 inhibit the ATPase and protein refolding capability of HSP-1

**Figure 2:**
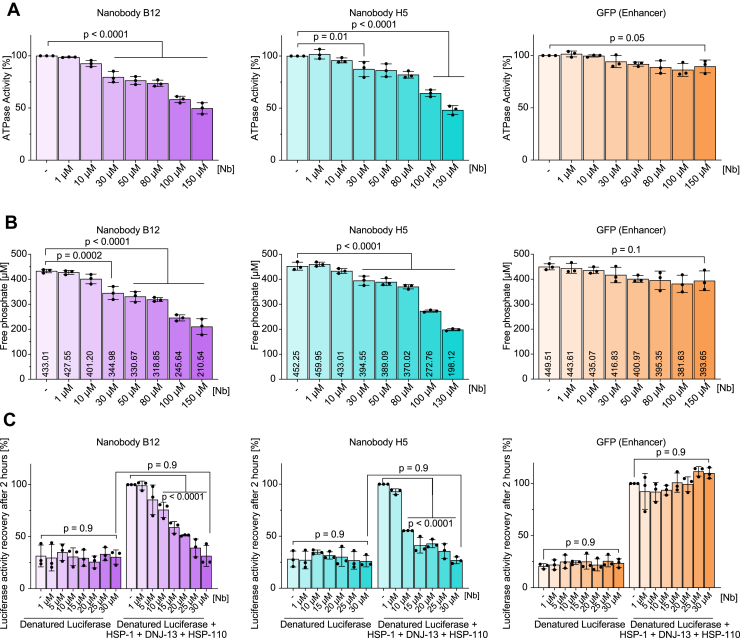
**Nanobodies B12 and H5 inhibit HSP-1 function *in vitro*.***A*, HSP-1-specific (*A*) ATPase assays and (*B*) quantifications of free-phosphate formation of reaction mixtures supplemented with indicated amount of nanobody B12, H5, or VHH Enhancer. Bars represent mean value of three replicates and error bars corresponds to the mean standard deviation. *C*, HSP-1-driven Luciferase refolding assay supplemented with indicated amount of nanobody B12, H5, or VHH Enhancer. Luciferase activity was measured following a 2-h recovery and normalized to the values measured for the Trimeric-chaperone complex sample without nanobody. Ordinary one-way ANOVA. *p* < 0.05 is considered statistically significant. VHH, variable domain of the heavy chain; HSP, heat shock protein.

After establishing B12 and H5 bind to HSP-1 *in vitro*, we next wondered if these nanobodies could be used to alter HSP-1 function. First, we sought to determine if B12 or H5 impacted the ATPase activity of HSP-1. *In vitro* ATPase assays showed that both B12 and H5 inhibited the DNJ-13-induced ATPase activity of HSP-1 in a dose-dependent manner. We observed significant reductions upon addition of 30 μM and a ∼50% reduction upon addition of 150 μM of either nanobody (Fig. 2, *A* and *B*). Addition of an anti-GFP nanobody did not affect ATP hydrolysis or free phosphate production, again suggesting this result is specific to B12 and H5 (Fig. 2, *A* and *B*).

Next, we assessed the ability of the nanobodies to affect refolding of denatured luciferase by HSP-1 and co-chaperones (DNJ-13, HSP-110) (26). Interestingly, the addition of low micromolar (10–30 μM) concentrations of either B12 or H5 into this reaction limited luciferase refolding in a dose-dependent manner, with complete inhibition between 20 to 30 μM (Fig. 2*C*). The concentration of the nanobodies required to inhibit HSP-1 function aligned well with the obtained binding constants (Fig. 1, *B* and *C*). However, the addition of the same concentrations of an anti-GFP (Enhancer) nanobody to the reaction mixture had no effect on luciferase refolding, suggesting this inhibition of HSP-1 refolding capability is specific to B12 and H5 (Fig. 2*C*).

Altogether, these data suggest B12 and H5 inhibit HSP-1 protein refolding capability and ATPase function *in vitro*.

### B12 and H5 bind to HSP-1 in complex *C. elegans* lysates

**Figure 3:**
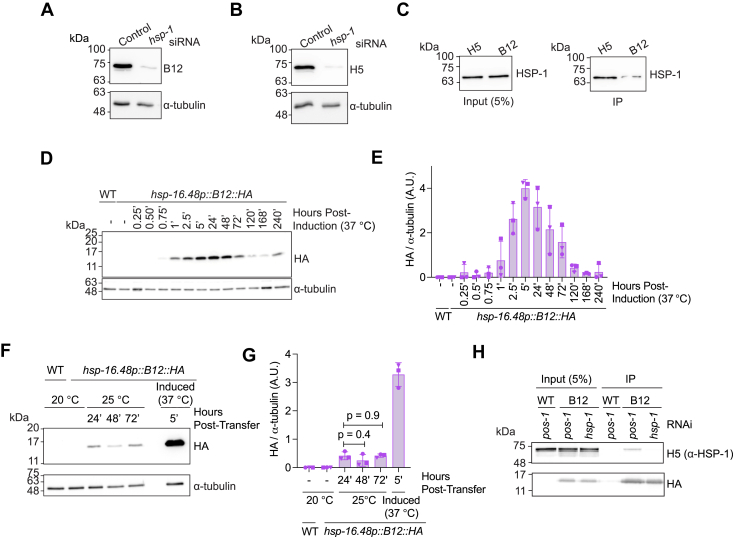
**B12 and H5 detect HSP-1 from *C. elegans’* lysate.***A* and *B*, western blots of day 2 *C. elegans* that were grown on control (*pos-1* RNAi) plates and then transferred to either *pos-1* or *hsp-1* RNAi plates for 24 h as day 1 adults. *pos-1* encodes a zinc-finger transcription factor required for embryonic development and sterilizes worms without affecting hatched animals (57, 58, 59, 60, 61, 62). Membranes were probed with either biotinylated (*A*) B12 or (*B*) H5 and α-tubulin (DSHB, Cat #12G10). *C*, western blots from immunoprecipitation assay using recombinant H5 or B12 as the primary antigen-binding agent and probed with an anti-HSC70 antibody (Proteintech, Cat #10654-1-AP) and α-tubulin (DSHB, Cat #12G10). *D* and *E*, example (*D*) western blot and (*E*) quantification of nanobody B12 expression (MTX298 (*hsp-16.48p::B12::HA*)) induced by a 30-min heat shock at 37 °C (HA, Cell Signaling (C29F4) and α-tubulin (DSHB, Cat #12G10)). *F* and *G*, example (*F*) western blot and (*G*) quantification of nanobody B12 expression (MTX298 (*hsp-16.48p::B12::HA*)) placed at 25 °C for indicated amount of time or induced by 30-min heat shock at 37 °C (HA, Cell Signaling (C29F4) and α-tubulin (DSHB, Cat #12G10). Induction was started in day 1 adults. *H*, co-immunoprecipitation assay of WT and MTX298 (“B12”) grown on indicated siRNA and collected 5 h post 30-min heat shock at 37 °C using magnetic anti-HA beads. Ordinary one-way ANOVA. *p* < 0.05 is considered statistically significant. HSP, heat shock protein; HA, hemagglutinin; HSC70, heat shock cognate 70.

Antibodies and nanobodies are most useful if they recognize target antigens in complex samples, including cell or tissue lysates. We thus sought to determine if B12 and/or H5 nanobodies recognize endogenously expressed HSP-1. Using B12 or H5 as the primary antigen-binding moiety to detect HSP-1 in lysates *via* Western blot, we found these nanobodies detected a single dominant band at the approximate molecular weight of HSP-1 (∼73 kDa) in wild-type *C. elegans* lysate but not in lysates of worms in which HSP-1 levels were depleted using *hsp-1* RNAi (Figs. 3, *A* and *B* and S2, *A*–*C*). We also observed an *hsp-1* siRNA-sensitive faint band at ∼70 kDa migrating slightly faster than the dominant band, suggesting that B12 and H5 detect distinct HSP-1 populations that likely vary in their posttranslational modification patterns (Figs. 3, *A* and *B* and S2, *A*–*C*). In subsequent immunoprecipitation (IP) assays, we confirmed that biotinylated H5 and, to a lesser extent, B12, captured HSP-1 from the soluble fraction of total *C. elegans* lysates (Fig. 3*C*). These results confirm B12 and H5 detect endogenous HSP-1 in complex environments.

### Inducible expression of HSP-1-specific nanobodies is tolerated and bind to HSP-1 *in vivo*

We next generated transgenic *C. elegans* strains expressing nanobodies B12, H5, and VHH-7 containing a C-terminal HA-tag under control of the heat-inducible *hsp-16.48* promoter. VHH-7, a nanobody specific for major histocompatibility complex class II (45), served as nontargeting nanobody control. A 30-min heat shock at 37 °C induced B12 expression, which peaked 5 h post induction and remained detectable for at least 10 days (Figs. 3, *D* and *E* and S3*A*), whereas continuous cultivation of worms at 25 °C, a condition known to induce modest heat stress, generated basal expression at approximately 16% of peak level (Figs. 3, *F* and *G* and S3*B*). We observed comparable expression patterns for nanobodies H5 and VHH-7, although the magnitude of expression varied depending on the specific nanobody-expressing strain and was strongest in the B12-expressing strain (Figs. S3, *C–E* and S6). These results demonstrate that camelid nanobodies can be efficiently expressed in transgenic *C. elegans*. Using magnetic agarose beads conjugated with anti-HA antibodies, we also showed that pulling on the HA-tag of B12 allowed for the co-immunoprecipitation of the B12-HSP-1 complex from worm lysates (Figs. 3*H* and S3*F*). Altogether, these data indicate that *in vivo* expressed B12 nanobodies interact with HSP-1.

### *In vivo* expression of B12, but not H5, reduces survival and proteotoxic stress resistance in *C. elegans*

**Figure 4:**
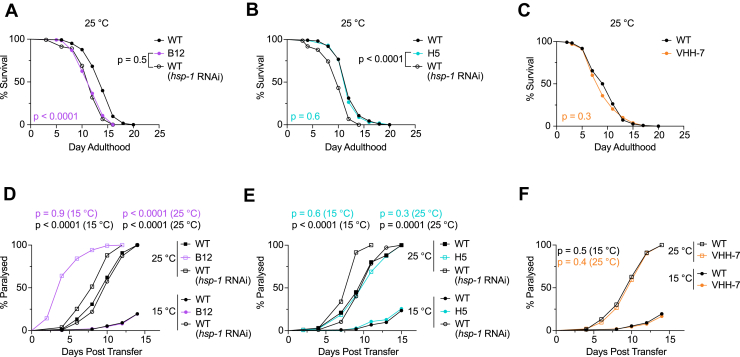
**Mild expression of B12 mimics phenotypes of *hsp-1* knockdown in *C. elegans*.***A*–*C*, survival curves of animals placed at 25 °C beginning on day 1 of adulthood. *D*–*F*, paralysis curves of Aβ_1–42_ -expressing animals at indicated temperature. For *A*–*C*, WT: wild type; B12: MTX298 (*hsp-16.48p::B12::HA*); H5: MTX277 (*hsp-16.48p::H5::HA*); VHH-7: MT24421 (*hsp-16.48p:: VHH-7::HA*). For *D*–*F*, WT: GMC101 (*dvIs100*); B12: MTX307 (*mtmIs20; dvIs100*),); H5: MTX319 (*mtmEx103; dvIs100*),; MTX329 (*nIs775; dvIs100*). Number of animals, median lifespan, and median day of paralysis are shown in Tables S2 and S3. Replicate experiments are shown in Figs. S4 and S5. Log-rank Mantel-Cox test. *p* < 0.05 is considered statistically significant. VHH, variable domain of the heavy chain; HSP, heat shock protein

Given that B12 and H5 inhibit HSP-1 chaperone function *in vitro* (Fig. 2) and that they recognize endogenous HSP-1 (Fig. 3), we sought to test if these nanobodies inhibit HSP-1 activity in living *C. elegans*. To express and sustain nanobody expression without inducing a severe heat stress, we transferred adult WT and nanobody-expressing animals to 25 °C and measured their survival. Low-level B12 expression at 25 °C significantly shortened adult survival, matching the reduction in survival observed when *hsp-1* was knocked down in WT animals using RNAi (Figs. 4*A* and S4*A*). In contrast, H5 or control-nanobody expressing animals kept at 25 °C did not exhibit a decrease in survival compared to WT (Figs. 4, *B* and *C* and S4, *B* and *C*). Survival was unchanged in B12 or H5-expressing animals following a 30-min induction at 37 °C compared to WT animals (Fig. S4, *D* and *E*).

We then wondered if nanobody expression altered the worm’s ability to mitigate protein misfolding stress. To test this prediction, we introduced the nanobody-encoding alleles into a strain expressing human Aβ_1–42_ in body-wall muscle cells (GMC101 (*dvIs100 [unc-54p:: Aβ*_*1–42*_*::unc-54 3′-UTR + mtl-2p::GFP])* (46)). When placed at the inducive temperature of 25 °C, these animals progressively lose motor function due to the accumulation of misfolded Aβ_1–42_^57^. We hypothesized that B12 and H5 expression would accelerate paralysis in a GMC101 background. Indeed, at 25 °C we observed B12-expressing animals paralyzed significantly faster than WT worms on both control and *hsp-1* siRNA at the same temperature (Figs. 4*D* and S5*A*). Interestingly, there was no difference in paralysis between B12 and WT animals at the noninducive temperature (15 °C), in which the *hsp-16.48* promoter is almost entirely inactive; this was unlike WT animals at the noninducive temperature on *hsp-1* siRNA, which showed significantly increased rates of paralysis (Figs. 4*D* and S5*A*). Notably, no comparable differences in paralysis were observed at both the noninducive and inducive temperatures in animals which expressed H5 or VHH-7 (Figs. 4, *E* and *F* and S5, *B* and *C*).

Collectively, these results demonstrate low-level expression of B12 effectively suppresses HSP-1 activity *in vivo* and phenocopies *hsp-1* knockdown. Our results further confirm the necessity of HSP-1 for mild heat stress survival and proteotoxic stress-resistance in *C. elegans*.

## Discussion

In this study, we identified and characterized two novel HSP-1-specific nanobodies (B12 and H5). We show that these nanobodies, which differ by only two amino acids in their CDR1 region, bind to HSP-1 both *in vitro* and *in vivo* (Figs. 1, and 3). Using HSP-1-specific ATPase and luciferase refolding assays, we show that B12 and H5 inhibit the ATPase activity, as well as the ability of HSP-1 to refold misfolded protein (luciferase), in a dose-dependent manner (Fig. 2). Finally, we show that low level *in vivo* expression of B12 (Fig. 3) shortens survival and reduces proteotoxic stress resistance in *C. elegans* —replicating the phenotypes of *hsp-1* knockdown (Fig. 4). Overall, these results demonstrate the effectiveness and specificity of these nanobodies to inhibit a specific HSP70 family chaperone *in vitro* and *in vivo*.

The ability to modulate HSP70 chaperone levels or activity is essential to understanding the physiological functions of these proteins in aging and protein-misfolding diseases. By precisely controlling HSP70 function in living organisms, we can begin to delineate the specific roles of HSP70 chaperones in disease processes and potentially develop novel targeted therapeutics. However, the ability to alter *in vivo* chaperone activity remains difficult, since many HSP70s are essential and thus hard to target using conventional genetic approaches. Furthermore, the conserved structural similarity (47) of HSP70 chaperones makes finding protein-specific small molecule modulators difficult to utilize. Thus, the temporal expression of nanobodies which effectively reduce the function of a specific HSP70 chaperone presents an exciting and effective strategy to advance our knowledge of the functions of specific chaperones in different physiological and pathophysiological contexts. Furthermore, while we did address nanobody specificity for different *C. elegans* and human proteins (Fig. 1), whether B12 or H5 may bind to other conserved HSP70 chaperones across species (*e.g.*, DnaK in *E. coli* or Ssa1 in *S. cerevisiae*) remains to be empirically determined.

Interestingly, we observed that, *in* *vivo*, B12 is more potent to reduce HSP-1 activity than H5. This discrepancy may at least in part be explained by differences in absolute nanobody levels upon transgene induction. Despite utilizing the same promoter to express B12, H5, and VHH-7 in *C. elegans*, transgene copy number, chromosomal position effects, partial epigenetic silencing, integrated *versus* extrachromosomal arrays, and differences in mRNA stability or translation efficiency likely prevent to achieve uniform transgene expression levels across strains (48).

Overall, our data demonstrate the effectiveness of using nanobodies to inhibit HSP70 family chaperone activity *in vitro* and *in vivo*.

### Study limitations

Our approach of expressing B12 and H5 under a stress-induced promoter is effective as it provides robust protein expression. However, it does limit the experimental design to assays which maintain nanobody expression under what could be considered “stressful” conditions (*e.g.*, elevated temperature); thus, interpretations as to the basal physiological roles of HSP-1 may be complex to interpret. In the future, this may be circumvented using other inducible technologies, such as tetracycline-inducible systems (*e.g.*, Tet/Q hybrid system) (49), optogenetic systems (50), or chemically induced systems (51, 52), which would allow for temporal expression without the need for a canonical mild stressor. However, these require the assembly of more complex transgenics and/or specialized equipment, thus limiting their ease of generation and use. Regardless, our results demonstrating B12 expression increases misfolding-induced paralysis at 25 °C but has no effect at the noninducive 15 °C (Fig. 4*D*) clearly shows the effectiveness of this current system while providing a base for future tool generation and expansion. Finally, K_D_ value measurements for nanobody B12 were confounded as the nanobody precipitated at concentrations above 100 μM, preventing us from testing nanobody-chaperone interactions at concentrations high enough to reach the assay’s plateau phase. The K_D_ value shared in the results section reflects a close approximation of the true K_D_ value.

## Experimental procedures

### Nanobody purification and labeling with sortase

Following a previously described protocol (53), nanobodies were expressed in the periplasm of *E. coli* cells, extracted following outer membrane rupture, and retrieved using Ni-NTA beads. Eluted nanobodies were dialyzed to remove excess imidazole. For labeling of nanobodies with biotin or TAMRA, we mixed 10 μg of sortase with a 20-fold molar excess of GGG-biotin or GGG-TAMRA and incubated the reaction at 4 °C overnight. The following day, we removed unconjugated dye and biotin molecules using P10 desalting columns and unconjugated nanobodies using Ni-NTA.

### Chaperone protein purification

All *C. elegans* chaperones were purified according to previously described protocols (54, 55). Subsequently, 100 ng of pSumo plasmids containing the chaperone sequences were transformed into *E. coli* BL21(DE3) (New England BioLabs, #C2527I). A starter culture was prepared by inoculating 30 ml of LB supplemented with Ampicillin (100 mg/ml) media with five transformants followed by incubation at 37 °C and shaking at 130 RPM overnight. The next morning, 2 L of LB-Ampicillin media were inoculated with 20 ml of the starter culture and incubated at 37 °C and shaking at 130 RPM. When *A*_600_ reached 0.6, protein expression was induced by adding 1 mM IPTG and continuing incubation at 20 °C with shaking at 130 rpm overnight. The next morning, bacteria were pelleted by centrifugation at 6000 rpm for 30 minutes at 4 °C. Pellet was thawed in ice and resuspended in 100 ml of lysis buffer (30 mM Hepes pH 7.4, 500 mM KAc, 5 mM MgCl_2_, 20 mM imidazole, 10% glycerol, 1 mM PMSF, 1 mM β-mercaptoethanol, 10 μg/ml DNase I, 1 tab/50 ml of cOmplete EDTA-free protease inhibitor cocktail (Roche)). The cell suspension was sonicated (Branson 450 Sonifier) for 10 min (20 s on; 40 s off) at 50% amplitude and soluble fraction was recovered after centrifugation at 16,000 rpm (Sorvall RC6+, Thermo Fisher Scientific) for 30 min at 4 °C. In total, 3 ml of Ni-NTA slurry (High-Density Nickel 6BCL-NTANi, Agarose Bead Technologies) were added to the soluble fraction, and His-tagged protein binding was allowed to occur at 4 °C with gentle rotation over a period of 1 hour. Slurry was filtered through a gravity flow column and washed with 25 ml of high-salt (30 mM Hepes pH 7.4, 1 M Kac, 5 mM MgCl_2_, 20 mM imidazole, 10% glycerol, and 1 mM β-mercaptoethanol) and low-salt (30 mM Hepes pH 7.4, 50 mM Kac, 5 mM MgCl_2_, 20 mM imidazole, 10% glycerol, and 1 mM β-mercaptoethanol) buffers. His-tagged proteins were recovered after addition of 4 ml of elution buffer (30 mM Hepes pH 7.4, 50 mM Kac, 5 mM MgCl_2_, 300 mM imidazole, 10% glycerol, and 1 mM β-mercaptoethanol) and incubation for 30 min at 4 °C with gentle rotation. Elution fraction was transferred to a 12 to 14 kDa MWCO dialysis membrane (Spectra/Por 2, Spectrum laboratories) and buffer was exchanged overnight at 4 °C against 2 L of dialysis buffer (30 mM Hepes pH 7.4, 50 mM Kac, 10% glycerol, and 1 mM β-mercaptoethanol) supplemented with 100 μl of 0.76 mg/ml His-Ulp1 protease to cleave the His-Smt3 tag. To remove the cleaved tag and the protease, protein solution was incubated with 1.5 ml of Ni-NTA slurry for 30 min at 4 °C and filtered through a gravity flow column. The recovered protein was aliquoted and flash-frozen in liquid nitrogen for storage at −80 °C.

### Microscale thermophoresis

Microscale thermophoresis experiments were performed in duplicate using a Monolith NT.115 instrument (Nanotemper Technologies). HSP-1 was labeled with RED-NHS using the Protein Labeling Kit RED-NHS second Generation (#MO-L011) following the manufacturer’s protocol. For binding assays, 20 nM of labeled HSP-1 was mixed with a 16-step serial titration of each binding partner and incubated for 30 min in the dark prior to measurement. For HSP-1:H5 binding experiments, concentrations ranged from 0.02664 to 436.5 μM; for HSP-1:B12, concentrations ranged from 0.003117 to 102.2 μM; and for HSP-1:Enhancer, the concentrations ranged from 0.0106 to 350 μM. Data were analyzed using MO.Control v2.7.1 software. Binding constant (K_d_) was determined using the built-in K_d_ model, and the binding curve was normalized as fraction bound.

### Luciferase refolding assays

Luciferase assay was performed as previously described with slight modifications (56). Briefly, a 3 nM luciferase solution in 1× dilution buffer (50 mM Hepes pH 7.4, 100 mM Kac, 5 mM MgCl_2_, 1 mM DTT, 10 μM BSA, 3.5 μM pyruvate kinase (Roche), 3 mM phosphoenol pyruvate) was denatured at 45 °C for 15 minutes. Next, luciferase was diluted to a final concentration of 1 nM in 1× dilution buffer containing 5 μM HSP-1, 0.25 μM HSP-110, and 5 μM DNJ-13 and amount of nanobody as described in the figure. After 2 h, 5 μl aliquots from each refolding sample were dispensed to three different wells of a 96-well white polystyrene plate (MultiScreen 96-Well-Plate, Millipore) containing 100 μl of assay buffer (25 mM glycylglycine, 100 mM Kac, 15 mM MgCl_2_, and 5 mM ATP). Then, 100 μl of 1 μM luciferin solution was added to each well, and luminescence was measured using a plate reader (Infinite 200 PRO, Tecan). Attenuation was not used, integration time was 1000 ms, and settle time was 0 ms. Values were normalized to the values measured for the Trimeric-chaperone complex sample after 2 h and data were presented as the percentage of luciferase activity recovered after 2 h.

### ATPase assays

ATPase assays were performed as previously descried with slight modifications (56) Briefly, 50 μl samples were prepared containing 1× reaction buffer (50 mM Hepes pH 7.4, 100 mM KAc, 5 mM MgCl_2_, and 0.017% Triton X-100), 5 μM HSP-1, 0.25 μM HSP-110, and 5 μM DNJ-13 and amount of nanobody as described in the figure. ATP was added to initiate the reaction at a final concentration of 2 mM, followed by incubation at 20 °C for 1 h. Ten microliter aliquots of phosphate standards and of each sample were transferred in triplicates to a 96-wells transparent microplate (Greiner), followed by 160 μl of green malachite reaction solution (2:1:3 dilution of 0.082% green malachite, 5.7% ammonium molybdate (in 6 M HCl) and water) and 20 μl of 34% sodium citrate. Absorbance at 650 nm was measured in a plate reader (Infinite 200 PRO, Tecan). To determine ATPase activity (%) for each sample, measurements were normalized to the activity of the Trimeric-chaperone complex. The corresponding free phosphate concentration in each well was calculated using the equation derived from the phosphate calibration curve.

### *C. elegans* strain preparation and maintenance

All worms were maintained at 15 °C on standard nematode growth media (NGM) plates spotted with OP50 to 1 *E. coli* for at least two generations without starving before being used for experiments. MTX265 (*mtmEx100[myo-2p::mCherry; hsp-16.48p:: B12::HA])* and MTX277 *mtmEx103[myo-2p::mCherry; hsp-16.48p:: H5::HA])* were generated by SUNY Biotech by microinjecting 10 ng/μl of marker and 10 ng/μl of transgene plasmid DNA into the germline of young adult N2 worms. *mtmEx100* was integrated *via* UV-irradiation to generate MTX298 (*mtmIs20[myo-3p::mCherry; hsp-16.48p:: B12*]) and backcrossed 5× to WT animals prior to being used in experiments. MT24068 (*nEx2480[myo-3p::mCherry; hsp-16.48p:: VHH-7::HA]* was generated by microinjection and integrated MT24421 (*nIs775[myo-3p::mCherry; hsp-16.48p:: VHH-7::HA]*). GMC101 (*dvIs100 [unc-54p::A-beta-1-42::unc-54 3′-UTR + mtl-2p::GFP]*) was obtained from the *Caenorhabditis* Genetics Center (CGC). MTX307 (*mtmIs20; dvIs100*), MTX319 (*mtmEx103; dvIs100*), and MTX329 (*nIs775; dvIs100*) were generated by the Truttmann Lab. Wild type (N2, Bristol) animals obtained from CGC and were used as reference controls unless otherwise stated. All strains were backcrossed at least three times before being used in experiments.

### Gene knockdown *via* RNA interference

RNA interference was performed as previously described (57). Briefly, HT115 *E. coli* expressing siRNA against the target gene of interest was grown in a 5× overnight culture of LB media. The following day, the overnight culture was spun down, and the pellet was resuspended in fresh 1× LB media (*e.g.* 5 ml of overnight culture resuspended in 1 ml of fresh LB). The 1× culture was supplemented with 100 mg/ml carbenicillin (1:1000, antibiotic, GoldBio, Cat #C-103-25) and 1M IPTG (1:200, Dot Scientific, Cat #DSI5600-25). The culture was then spotted on to NGM plates that were supplemented with 1M IPTG (1:1000), 100 mg/ml carbenicillin (1:1000) and 10 mg/ml nystatin (1:1000, antifungal, Dot Scientific, Cat #DSN82020-10). Plates were used immediately on the day of preparation or kept at 4 °C for no more than 2 to 3 days. For all experiments, worms were synchronized onto HT115 *E. coli* expressing siRNA against *pos-1*, which is a zinc-finger transcription factor required for embryonic development and sterilizes worms without affecting the hatched animal (57, 58, 59, 60, 61, 62). All siRNA-expressing *E. coli* were obtained from the Vidal Library (63) and validated for target specificity previously (25, 62).

### Worm synchronization

Worms were synchronized *via* hypochlorite treatment as previously described (57). Briefly, animals were washed 3× with sterile filtered M9 and treated with 1 ml of hypochlorite solution. Animals were incubated for 8 to 10 min with shaking at room temperature. Following bleaching, eggs were pelleted using centrifugation (2900 rcf) and washed 2× with sterile M9 before being plated.

### Worm lysis for protein biochemistry

Worms were lysed as previously described (57). Briefly, worms were washed 3× with sterile M9 buffer and snap frozen in liquid nitrogen then stored at −80 °C. Samples were resuspended in ∼200 to 400 μl of sterile filtered worm lysis buffer (Hepes (20 mM, 7.4 pH), NaCl (20 mM), MgCl_2_ (200 mM), and Nonidet P-40 (0.5%)) spiked with protease inhibitor cocktail (Pierce Protease and Phosphatase Inhibitor Mini Tablets, EDTA-free, Thermo Fisher Scientific, Cat #A32961). Worms were transferred to reinforced tubes with a steel ball and lysed using a Qiagen TissueLyser III (7.5 min, 30 Hz). Lysate was cleared at 16,100 rcf (4 °C, 15 min) twice, transferring to a precooled tube after each transfer. The soluble fraction was collected, and protein lysate concentration was determined using the Pierce BCA Protein Assay Kit (Thermo Fisher Scientific, Cat #23227) following the manufacture’s instruction.

### Immunoblotting

Either 1 μg of recombinant purified protein or 10 to 20 μg of *C. elegans* worm lysate was added to 4× Laemmli Protein Sample Buffer (Bio-Rad, Cat #1610747) as described by the manufacturer, then boiled at 100 °C for 5 min. Samples were subjected to SDS-PAGE and subsequently transferred to a polyvinylidene fluoride (PVDF) membrane using the Bio-Rad Trans-Blot Turbo System (Bio-Rad, Cat #1704150) and Trans-Blot Turbo RTA Transfer Kit, PVDF (Bio-Rad, Cat #1704272) following the manufacturer’s instruction. Membranes were blocked for 1 h at room temperature with gently rocking and probed with the appropriate antibody or nanobody (1 μg/ml) overnight at 4 °C with gentle rocking. Membranes were washed 3× with sterile 0.1% TBS-Tween 20 (TBS-T) and incubated with appropriate secondary antibodies for 1 h at room temperature while rocking. The membrane was then washed 3× with TBS-T. Table S1 lists all blocking buffers and antibodies used in this study. Chemiluminescent signal was observed using Prometheus Protein Biology ProSignal Dura ECL Reagent (Prometheus Protein Biology Products, Genesee Scientific, Cat #20–301) following the manufactures instructions. Membranes were imaged using an Invitrogen iBright1500. If necessary, membrane stripping was done using OneMinute Western Blot Stripping Buffer (GM Biosciences, Cat #GM6001) following the manufacturer’s instruction, washed vigorously with ddH_2_O, then rehydrated in 0.1% TBS-T for 15 min. Membranes were then treated as described above. Immunoblot quantification was done using Fiji (version 2.14).

### Immunoprecipitation assays

Worms were synchronized and lysed as described above. Magnetic protein G agarose beads were washed with 300 μl of worm lysis buffer and separated using a magnetic rack 3 times (Dynabeads Protein G for Immunoprecipitation, Invitrogen, Cat. #10003). The appropriate amount of worm lysate (∼500–1000 μg) was diluted to an equal volume of worm lysis buffer, then precleared for at least 1 h at 4 °C while rotating using washed beads. Following clearing, an aliquot was saved to use as input control (5% of total immunoprecipitation). For immunoprecipitation using exogenous nanobody, 20 μl/sample of preconjugated streptavidin magnetic beads (Dynabeads MyOne Streptavidin C1, Thermo Fisher Scientific, Cat. #65001) were washed three times and added to the precleared lysate. For immunoprecipitation of nanobodies from *in vivo* expression, 20 μl/sample of preconjugated anti-HA magnetic beads (Pierce Anti-HA Magnetic Beads, Thermo Fisher Scientific, Cat. #88836) were washed three times and added to the precleared lysate. Lysate/bead mixture was incubated over night at 4 °C with rocking. The following day, the protein-bead complex was isolated using a magnetic rack and washed 3× with cold worm lysis buffer. Following the final wash, the complex was resuspended in ∼15 to 30 uL of 4× Laemmli Protein Sample Buffer (Bio-Rad, Cat #1610747) as described by the manufacturer and boiled at 100 °C for 5 min. Beads were then isolated using a magnetic rack, and the supernatant was collected and subjected to SDS-PAGE and treated as described above (Immunoblotting).

### *C. elegans* mild heat stress survival assays

Worms were synchronized *via* hypochlorite treatment as previously described above. Eggs were spotted onto NGM/RNAi interference plates described in RNA interference above with HT115 *E. coli* expressing siRNA against *pos-1*. Worms were then placed in a 20 °C incubator. Day 1 adults were transferred to 60 mm IPTG-NGM plates spotted with fresh HT115 with siRNA targeting *pos-1* or *hsp-1* (∼40–50 worms per plate). Animals were then transferred to a 25 °C incubator. For heat shock survival assays, animals were transferred to a 37 °C incubator (induced) for 30 min. Plates were left for 1 hour to return to room temperature, then placed at 20 °C. Dead animals, as confirmed by lack of spontaneous or prodded movement, were removed from the plate and counted every other day until the completion of the experiment.

### Paralysis assays

Paralysis assays were performed as previously described (57). Briefly, animals were synchronized *via* hypochlorite treatment, then maintained at 15 °C until day 1 of adulthood. Animals were then transferred to fresh 60 mm IPTG-NGM plates spotted with fresh HT115 with siRNA targeting *pos-1* or *hsp-1* (∼40–50 worms per plate), then placed at either 25 °C or 15 °C. Animals were considered paralyzed if unable to complete a full body movement spontaneously or when prodded. Paralyzed animals were counted and removed from the plate when scored every other day.

## Data availability

The unprocessed raw datasets generated and analyzed during the current study are available from the corresponding author upon reasonable request.

## Supporting information

This article contains supporting information.

## Conflict of interest

The authors declare that they have no conflicts of interest with the contents of this article.

## References

[1] Yu E.M., Yoshinaga T., Jalufka F.L., Ehsan H., Mark Welch D.B., Kaneko G. (2021). The complex evolution of the metazoan HSP70 gene family. Sci. Rep..

[2] Flaherty K.M., DeLuca-Flaherty C., McKay D.B. (1990). Three-dimensional structure of the ATPase fragment of a 70K heat-shock cognate protein. Nature.

[3] Zhu X., Zhao X., Burkholder W.F., Gragerov A., Ogata C.M., Gottesman M.E. (1996). Structural analysis of substrate binding by the molecular chaperone DnaK. Science.

[4] Swain J.F., Schulz E.G., Gierasch L.M. (2006). Direct comparison of a stable isolated Hsp70 substrate-binding domain in the empty and substrate-bound states. J. Biol. Chem..

[5] Oshiro N., Takahashi R., Yoshino K.I., Tanimura K., Nakashima A., Eguchi S. (2007). The proline-rich Akt substrate of 40 kDa (PRAS40) is a physiological substrate of Mammalian target of Rapamycin complex 1. J. Biol. Chem..

[6] Mayer M.P., Bukau B. (2005). Hsp70 chaperones: cellular functions and molecular mechanism. Cell Mol. Life Sci..

[7] Rohland L., Kityk R., Smalinskaitė L., Mayer M.P. (2022). Conformational dynamics of the Hsp70 chaperone throughout key steps of its ATPase cycle. Proc. Natl. Acad. Sci. U. S. A..

[8] Kampinga H.H., Craig E.A. (2010). The HSP70 chaperone machinery: j proteins as drivers of functional specificity. Nat. Rev. Mol. Cell Biol..

[9] Misselwitz B., Staeck O., Rapoport T.A. (1998). J proteins catalytically activate Hsp70 molecules to trap a wide range of peptide sequences. Mol. Cell.

[10] Karzai A.W., McMacken R. (1996). A bipartite signaling mechanism involved in DnaJ-mediated activation of the Escherichia coli DnaK protein. J. Biol. Chem..

[11] Gupta A., Bansal A., Hashimoto-Torii K. (2020). HSP70 and HSP90 in neurodegenerative diseases. Neurosci. Lett..

[12] Witt S.N. (2010). Hsp70 molecular chaperones and Parkinson’s disease. Biopolymers.

[13] Turturici G., Sconzo G., Geraci F. (2011). Hsp70 and its molecular role in nervous system diseases. Biochem. Res. Int..

[14] Murphy M.E. (2013). The HSP70 family and cancer. Carcinogenesis.

[15] Abe M., Manola J.B., Oh W.K., Parslow D.L., George D.J., Austin C.L., Kantoff P.W. (2004). Plasma levels of heat shock protein 70 in patients with prostate cancer: a potential biomarker for prostate cancer. Clin. Prostate Cancer.

[16] Bobkova N.V., Garbuz D.G., Nesterova I., Medvinskaya N., Samokhin A., Alexandrova I. (2013). Therapeutic effect of exogenous Hsp70 in mouse models of alzheimer’s disease. J. Alzheimers Dis..

[17] Radons J. (2016). The human HSP70 family of chaperones: where do we stand?. Cell Stress Chaperones.

[18] Hartl F.U., Hayer-Hartl M. (2002). Molecular chaperones in the cytosol: from nascent chain to folded protein. Science.

[19] Brocchieri L., Conway De Macario E., Macario A.J. (2008). hsp70 genes in the human genome: conservation and differentiation patterns predict a wide array of overlapping and specialized functions. BMC Evol. Biol..

[20] Liu T., Daniels C.K., Cao S. (2012). Comprehensive review on the HSC70 functions, interactions with related molecules and involvement in clinical diseases and therapeutic potential. Pharmacol. Ther..

[21] Dastoor Z., Dreyer J.L. (2000). Nuclear translocation and aggregate formation of heat shock cognate protein 70 (Hsc70) in oxidative stress and apoptosis. J. Cell Sci..

[22] Tsukahara F., Maru Y. (2004). Identification of novel nuclear export and nuclear localization-related signals in human heat shock cognate protein 70. J. Biol. Chem..

[23] Bański P., Mahboubi H., Kodiha M., Shrivastava S., Kanagaratham C., Stochaj U. (2010). Nucleolar targeting of the chaperone hsc70 is regulated by stress, cell signaling, and a composite targeting signal which is controlled by autoinhibition. J. Biol. Chem..

[24] Wang F., Bonam S.R., Schall N., Kuhn L., Hammann P., Chaloin O. (2018). Blocking nuclear export of HSPA8 after heat shock stress severely alters cell survival. Sci. Rep..

[25] Truttmann M.C., Pincus D., Ploegh H.L. (2018). Chaperone AMPylation modulates aggregation and toxicity of neurodegenerative disease-associated polypeptides. Proc. Natl. Acad. Sci. U. S. A..

[26] Kirstein J., Arnsburg K., Scior A., Szlachcic A., Guilbride D.L., Morimoto R.I. (2017). *In vivo* properties of the disaggregase function of J-proteins and Hsc70 in Caenorhabditis elegans stress and aging. Aging Cell.

[27] Ciechanover A., Kwon Y.T. (2017). Protein quality control by molecular chaperones in neurodegeneration. Front. Neurosci..

[28] Kanack A.J., Olp M.D., Newsom O.J., Scaglione J.B., Gooden D.M., McMahon K. (2022). Chemical regulation of the protein quality control E3 ubiquitin ligase C-Terminus of Hsc70 interacting protein (CHIP). Chembiochem.

[29] Abildgaard A.B., Voutsinos V., Petersen S.D., Larsen F.B., Kampmeyer C., Johansson K.E. (2023). HSP70-binding motifs function as protein quality control degrons. Cell Mol. Life Sci..

[30] Rebeaud M.E., Mallik S., Goloubinoff P., Tawfik D.S. (2021). On the evolution of chaperones and cochaperones and the expansion of proteomes across the Tree of life. Proc. Natl. Acad. Sci. U. S. A..

[31] Hamers-Casterman C., Atarhouch T., Muyldermans S., Robinson G., Hamers C., Songa E.B. (1993). Naturally occurring antibodies devoid of light chains. Nature.

[32] Liu X., Wang Y., Sun L., Xiao G., Hou N., Chen J. (2024). Screening and optimization of shark nanobodies against SARS-CoV-2 spike RBD. Antivir. Res.

[33] Spinelli S., Frenken L., Bourgeois D., de Ron L., Bos W., Verrips T. (1996). The crystal structure of a llama heavy chain variable domain. Nat. Struct. Mol. Biol..

[34] Desmyter A., Transue T.R., Ghahroudi M.A., Thi M.H., Poortmans F., Hamers R. (1996). Crystal structure of a camel single-domain VH antibody fragment in complex with lysozyme. Nat. Struct. Mol. Biol..

[35] Chen W.H., Hajduczki A., Martinez E.J., Bai H., Matz H., Hill T.M. (2023). Shark nanobodies with potent SARS-CoV-2 neutralizing activity and broad sarbecovirus reactivity. Nat. Commun..

[36] Zhu H., Ding Y. (2025). Nanobodies: from discovery to AI-Driven design. Biology (Basel).

[37] Arbabi Ghahroudi M., Desmyter A., Wyns L., Hamers R., Muyldermans S. (1997). Selection and identification of single domain antibody fragments from camel heavy-chain antibodies. FEBS Lett..

[38] Zhou X., Hao R., Chen C., Su Z., Zhao L., Luo Z. (2020). Rapid delivery of Nanobodies/VHHs into living cells via expressing in vitro-transcribed mRNA. Mol. Ther. Methods Clin. Dev..

[39] Zhang J., Sun H., Pei W., Jiang H., Chen J. (2021). Nanobody-based immunosensing methods for safeguarding public health. J. Biomed. Res..

[40] Cater J.H., El Salamouni N.S., Mansour G.H., Hutchinson S., Mc Guinness C., Mueller S.H. (2025). Optimised nanobody-based quenchbodies for enhanced protein detection. Commun. Biol..

[41] Teunissen A.J.P., Abousaway O.B., Munitz J., van Leent M.M.T., Toner Y.C., Priem B. (2021). Employing nanobodies for immune landscape profiling by PET imaging in mice. STAR Protoc..

[42] Zhao S., Luo J., Xu P., Zeng J., Yan G., Yu F. (2025). Designed peptide binders and nanobodies as PROTAC starting points for targeted degradation of PCNA and BCL6. Int. J. Biol. Macromol..

[43] Kirchhofer A., Helma J., Schmidthals K., Frauer C., Cui S., Karcher A. (2010). Modulation of protein properties in living cells using nanobodies. Nat. Struct. Mol. Biol..

[44] Antos J.M., Ingram J., Fang T., Pishesha N., Truttmann M.C., Ploegh H.L. (2017). Site-specific protein labeling via sortase-mediated transpeptidation. Curr. Protoc. Protein Sci..

[45] Fang T., Duarte J.N., Ling J., Li Z., Guzman J.S., Ploegh H.L. (2016). Structurally defined αMHC-II nanobody-drug conjugates: a therapeutic and imaging system for B-Cell lymphoma. Angew. Chem. Int. Ed. Engl..

[46] McColl G., Roberts B.R., Pukala T.L., Kenche V.B., Roberts C.M., Link C.D. (2012). Utility of an improved model of amyloid-beta (Aβ1-42) toxicity in Caenorhabditis elegans for drug screening for Alzheimer’s disease. Mol. Neurodegener..

[47] Hunt C., Morimoto R.I. (1985). Conserved features of eukaryotic hsp70 genes revealed by comparison with the nucleotide sequence of human hsp70. Proc. Natl. Acad. Sci. U. S. A..

[48] Frøkjaer-Jensen C., Davis M.W., Hopkins C.E., Newman B.J., Thummel J.M., Olesen S.P. (2008). Single-copy insertion of transgenes in Caenorhabditis elegans. Nat. Genet..

[49] Mao S., Qi Y., Zhu H., Huang X., Zou Y., Chi T. (2019). A Tet/Q hybrid system for robust and versatile control of transgene expression in C. elegans. iScience.

[50] Shin Y., Berry J., Pannucci N., Haataja M.P., Toettcher J.E., Brangwynne C.P. (2017). Spatiotemporal control of intracellular phase transitions using light-activated optoDroplets. Cell.

[51] Zhang L., Ward J.D., Cheng Z., Dernburg A.F. (2015). The auxin-inducible degradation (AID) system enables versatile conditional protein depletion in C. elegans. Development.

[52] Ashley G.E., Duong T., Levenson M.T., Martinez M.A.Q., Johnson L.C., Hibshman J.D. (2021). An expanded auxin-inducible degron toolkit for Caenorhabditis elegans. Genetics.

[53] Truttmann M.C., Wu Q., Stiegeler S., Duarte J.N., Ingram J., Ploegh H.L. (2015). HypE-specific nanobodies as tools to modulate HypE-mediated target AMPylation. J. Biol. Chem..

[54] Nillegoda N.B., Kirstein J., Szlachcic A., Berynskyy M., Stank A., Stengel F. (2015). Crucial HSP70 co-chaperone complex unlocks metazoan protein disaggregation. Nature.

[55] Scior A., Buntru A., Arnsburg K., Ast A., Iburg M., Juenemann K. (2018). Complete suppression of Htt fibrilization and disaggregation of Htt fibrils by a trimeric chaperone complex. EMBO J..

[56] Ayala Mariscal S.M., Pigazzini M.L., Richter Y., Özel M., Grothaus I.L., Protze J. (2022). Identification of a HTT-specific binding motif in DNAJB1 essential for suppression and disaggregation of HTT. Nat. Commun..

[57] Urban N.D., Lacy S.M., Van Pelt K.M., Abdon B., Mattiola Z., Klaiss A. (2025). Functionally diversified Caenorhabditis elegans Bip Orthologs control body growth, reproduction, stress resistance, aging, and autophagy. Nat Commun..

[58] Tabara H., Hill R.J., Mello C.C., Priess J.R., Kohara Y. (1999). pos-1 encodes a cytoplasmic zinc-finger protein essential for germline specification in C. elegans. Development.

[59] Tabara H., Sarkissian M., Kelly W.G., Fleenor J., Grishok A., Timmons L. (1999). The rde-1 gene, RNA interference, and transposon silencing in C. elegans. Cell.

[60] Farley B.M., Pagano J.M., Ryder S.P. (2008). RNA target specificity of the embryonic cell fate determinant POS-1. RNA.

[61] Taylor M.N., Spandana B.S., Vega N.M. (2022). Using single-worm data to quantify heterogeneity in caenorhabditis elegans-Bacterial interactions. J. Vis. Exp..

[62] Van Pelt K.M., Truttmann M.C. (2025). Loss of FIC-1-mediated AMPylation activates the UPR^ER^ and upregulates cytosolic HSP70 chaperones to suppress polyglutamine toxicity. PLoS Genet..

[63] Rual J.F., Ceron J., Koreth J., Hao T., Nicot A.S., Hirozane-Kishikawa T. (2004). Toward improving Caenorhabditis elegans phenome mapping with an ORFeome-based RNAi library. Genome Res..

