# Supplementary material for: HSP-1-specific nanobodies alter chaperone function in vitro and in vivo
Source: J Biol Chem. 2026 Feb 4;302(3):111238. doi: 10.1016/j.jbc.2026.111238 (PMC12966735; doi:10.1016/j.jbc.2026.111238)
Supplement: Supplemental Figure [file mmc2.pdf]

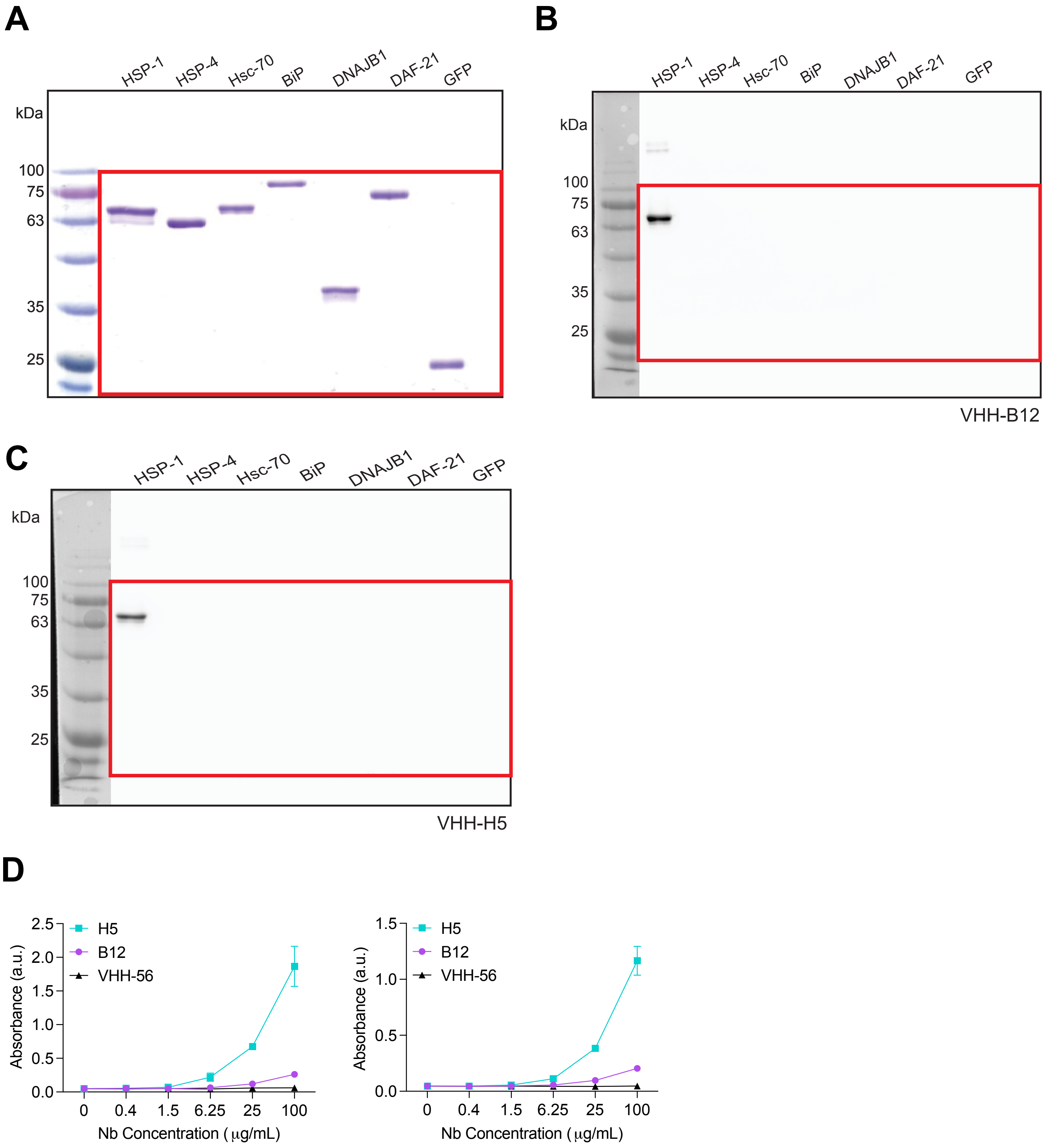

Supplemental Figure 1. B12 and H5 recognize HSP-1 in Western blots and ELISA assays. (A-C) Uncropped Coomassie stain and western blot example of blots shown in Figure 1. (D). Two additional replicates of ELISA assays using HSP-1 as the bait protein. Nanobodies were conjugated with biotin using sortase technology and detected using the Precision Protein StrepTactin-HRP Conjugate (BioRad, Cat #161038). Red box indicates what is shown in main text.

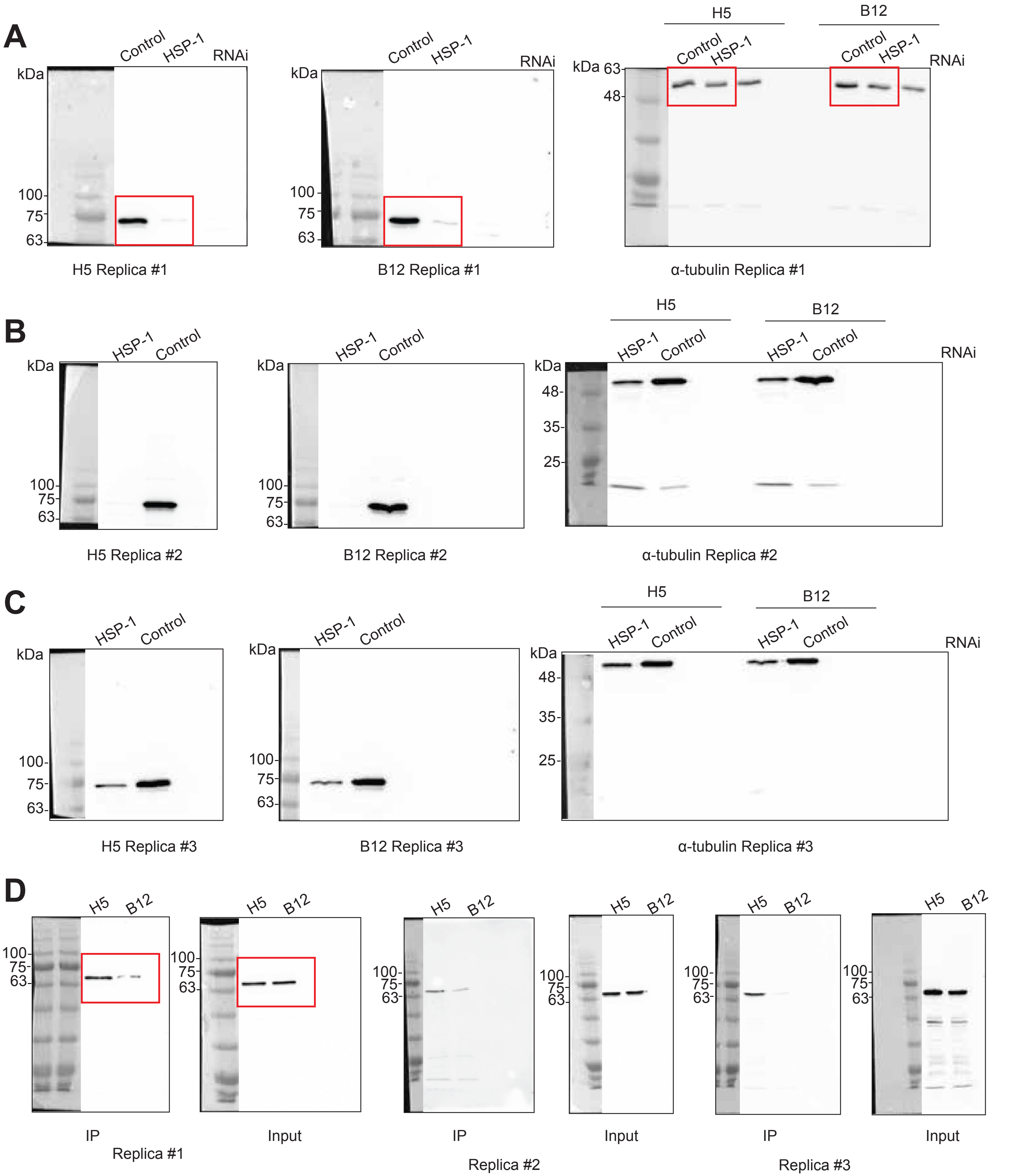

Supplemental Figure 2. B12 and H5 recognize endogenous HSP-1. (A-C). Three biological replicates of western blots showing biotinylated B12 and H5 detect HSP-1 in worm lysate. (D). Three biological replicates of western blots from immunoprecipitation assays using recombinant H5 or B12 as the primary antigen-binding agent. Membranes were probed with an anti-HSC70 antibody (Proteintech, Cat #10654-1-AP) and anti-α-tubulin (DSHB, Cat #12G10). Red box indicates what is shown in Figure 3.

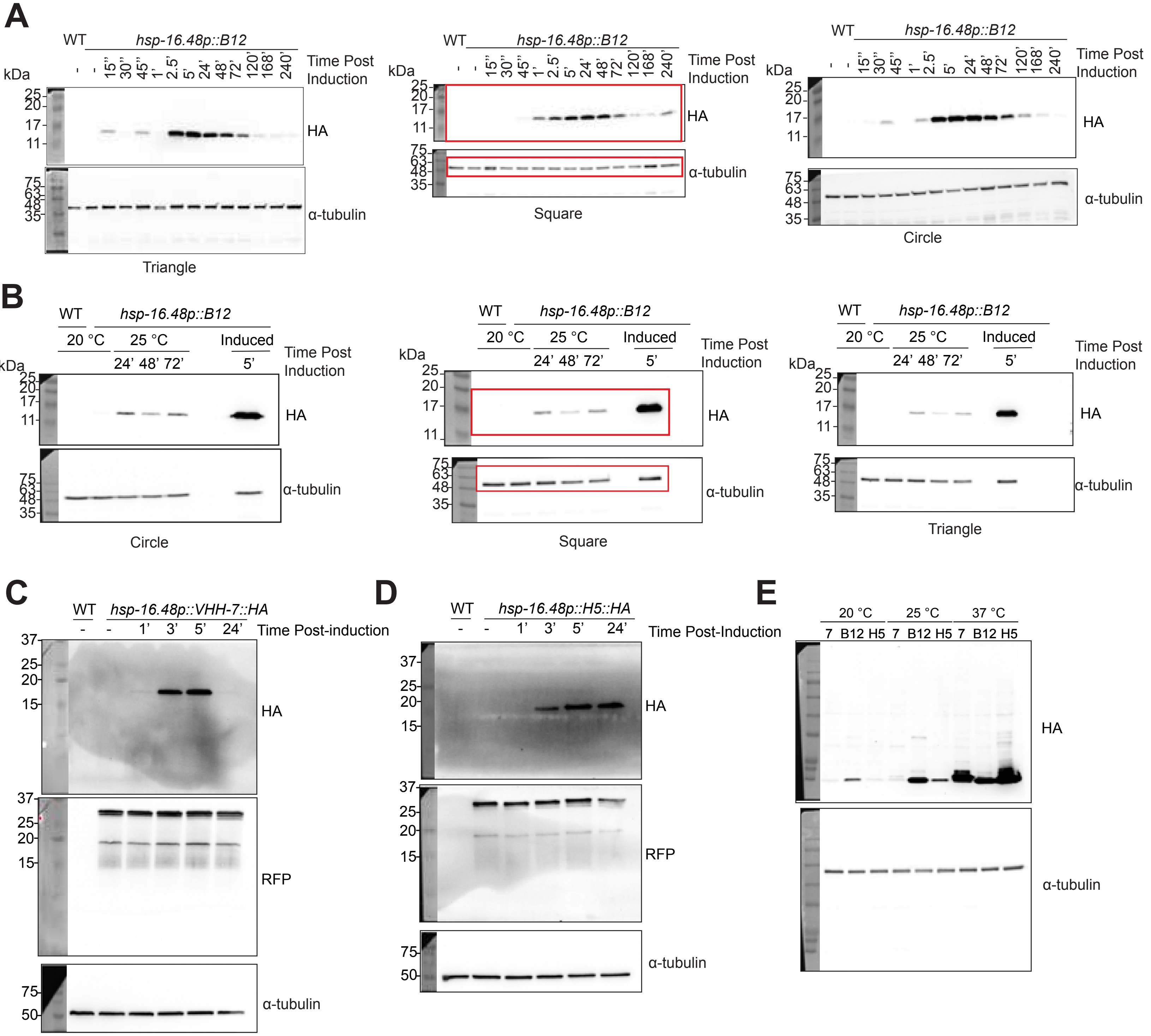

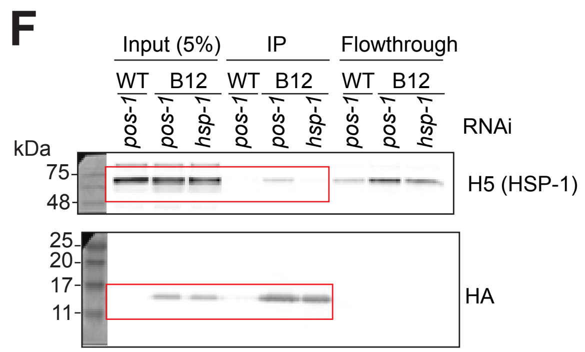

Supplemental Figure 3. HSP-1-specific nanobodies can be expressed endogenously in *C. elegans*. (A). Western blot replicates of time course assay for MTX298 (*hsp-16.48p::B12::HA*). Protein induction was induced by a 30-minute heat shock at 37 °C. (B). Western blot replicates of time course assay of MTX298 (*hsp-16.48p::B12::HA*) protein collected after placing day 1 adult animals at 25 °C for indicated amount of time or 5 hours after a 30-minute heat shock at 37 °C ("Induced"). (C-D). Western blots for protein expression time course assays of (C) MTX277 (*hsp-16.48p::H5::HA*) and (D) MT24421 (*hsp-16.48p::VHH-7::HA*). Nanobody induction was driven by a 30-minute heat shock at 37 °C. (E). Western blot comparing induction of MTX298 (B12), MTX277 (H5) and MT24421 (7) at indicated temperatures. Animals were synchronized and eggs were placed directly at either 20 °C or 25 °C and collected 72 hours later or induced as day 1 adults by a 30-minute heat shock at 37 °C and collected after 5 hours. (F). Uncropped immunoprecipitation assay of day 2 adult worms collected 5 hours after induction. Day 1 adults were transferred to either *pos-1* or *hsp-1* siRNA 24 hours prior to induction. "B12" is MTX298. HA, Cell Signaling (Cat #C29F4); RFP Proteintch (Cat #6g6), α-tubulin (DSHB, Cat #12G10). Red box indicates what is shown in Figure 3.

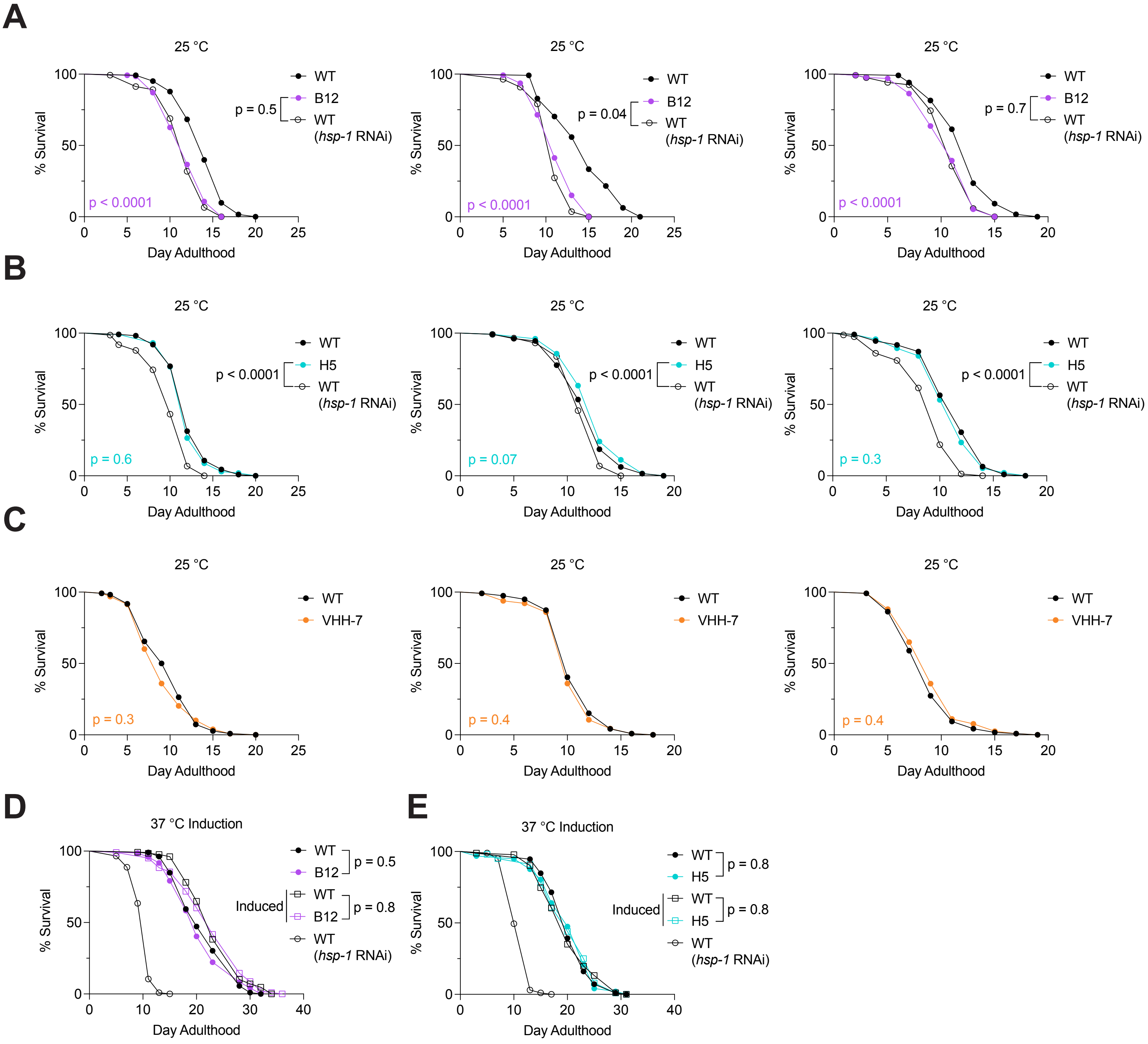

Supplemental Figure 4. Survival curves. (A-C). Replicates of survival curves for (A) MTX298, (B) MTX277, and (C) MT24421 which were grown for 72 hours at 20 °C then transferred to 25 °C as day 1 adults. (D-E). Survival curves of (D) MTX298 and (E) MTX277 following a 30-minute induction at 37 °C. Day 1 adult animals were induced. For all experiments WT animals were transferred to *hsp-1* siRNA as day 1 adults. WT: wild type; B12: MTX298 (*hsp-16.48p::B12::HA*); H5: MTX277 (*hsp-16.48p::H5::HA*); VHH-7: MT24421 (*hsp-16.48p:: VHH-7::HA*). *Log-rank Mantel-Cox test. p < 0.05 is considered statistically significant.* Median lifespan and number of animals for all experiments are listed in Supplemental Table 2.

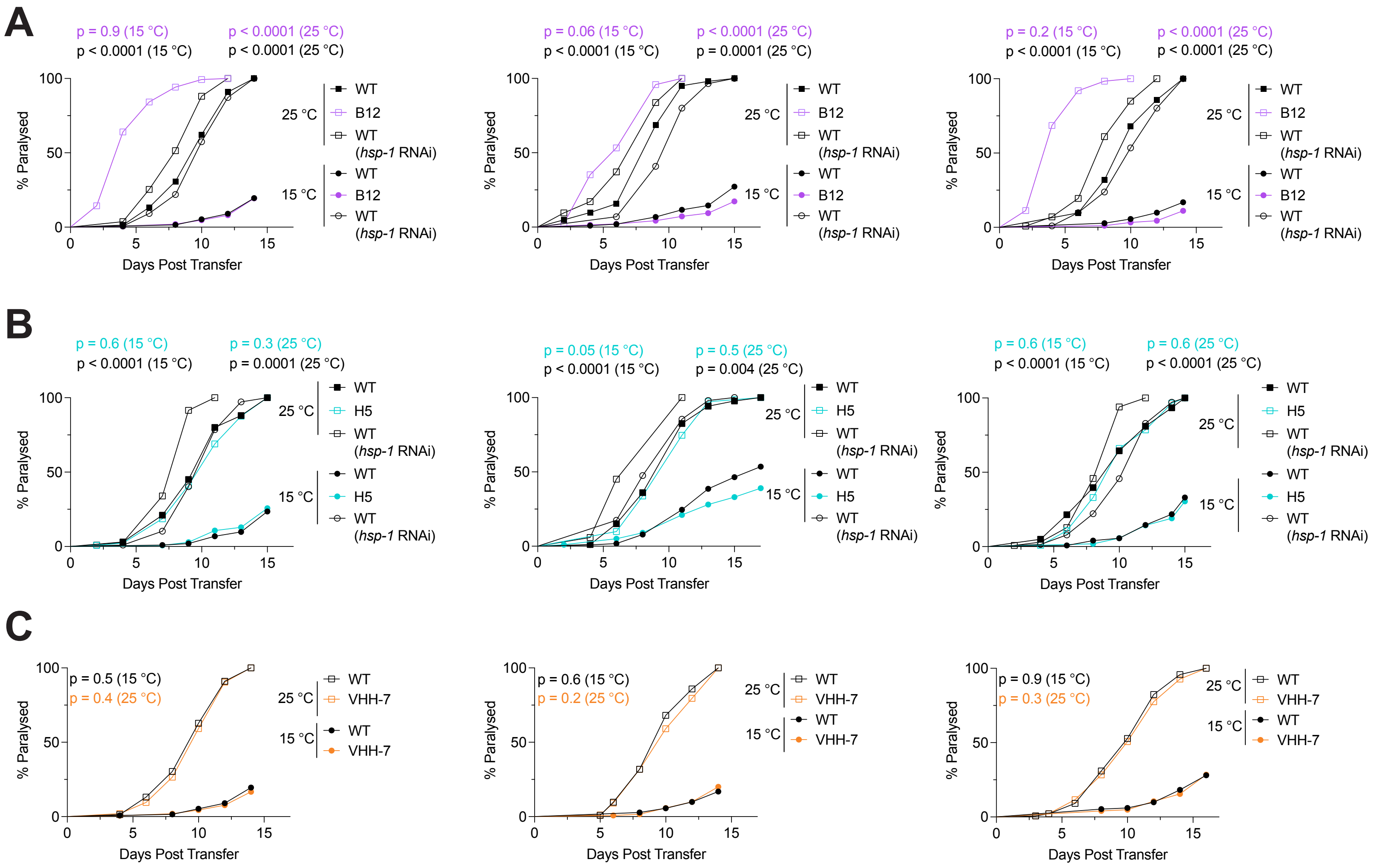

Supplemental Figure 5. Paralysis assays. (A-C). Replicates of paralysis assays for (A) MTX307 ("B12", *hsp-16.48p::B12::HA; dvIs100*), (B) MTX320 ("H5", *hsp-16.48p::H5::HA; dvIs100*), and (C) MTX329 ("VHH-7", *hsp-16.48p::VHH-7::HA; dvIs100*) which were grown for 72 hours at 20 °C then transferred to 25 °C as day 1 adults. WT: GMC101 (*dvIs100 [unc-54p::A-beta-1-42::unc-54 3'-UTR + mtl-2p::GFP]*). Log-rank Mantel-Cox test. *p < 0.05 is considered statistically significant.* Median paralysis and number of animals per experiment is listed in Supplemental Table 3.

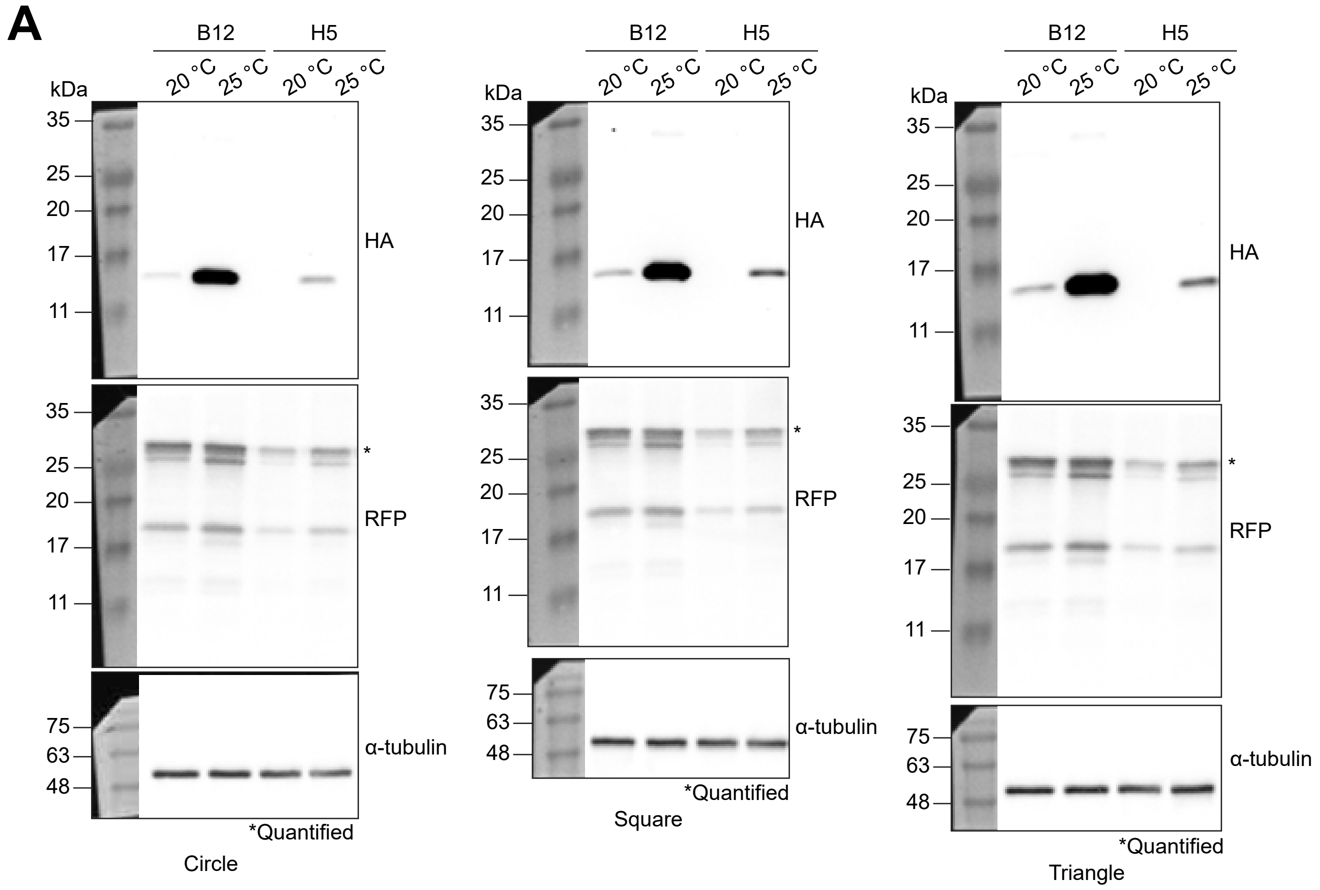

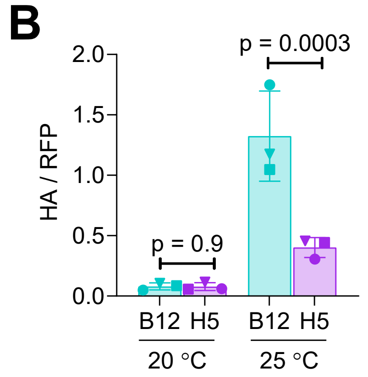

Supplementary Figure 6. Induction of B12 is more robust than H5. (A). Three biological replicates of day 1 adult MTX298 ("B12"; *hsp-16.48::B12::HA*) and MTX277 ("H5", *hsp-16.48::B12::HA*) placed at 20 °C or 25 °C for 24 hours. (B). Quantification of nanobody expression (HA) relative to co-expression marker (RFP). *2-way ANOVA followed by Uncorrected Fisher's LSD.* $p_{temperature\ x\ genotype} = 0.003$; $p_{temperature} = 0.0001$; $p_{genotype} = 0.003$
